# Band Alignment of Stacked Crystalline Si/GaN pn Heterostructures Interfaced with an Amorphous Region Using X-Ray Photoelectron Spectroscopy

**DOI:** 10.3390/ma17246099

**Published:** 2024-12-13

**Authors:** Kwangeun Kim

**Affiliations:** School of Electronics and Information Engineering, Korea Aerospace University, Goyang 10540, Republic of Korea; kke@kau.ac.kr; Tel.: +82-2-300-0139

**Keywords:** stacking, heterostructure, band alignment, nanomembrane, X-ray photoelectron spectroscopy

## Abstract

The energy band alignment of a stacked Si/GaN heterostructure was investigated using X-ray photoelectron spectroscopy (XPS) depth profiling, highlighting the influence of the amorphous interface region on the electronic properties. The crystalline Si/GaN pn heterostructure was formed by stacking a Si nanomembrane onto a GaN epi-substrate. The amorphous layer formed at the stacked Si/GaN interface altered the energy band of the stacked heterostructure and affected the injection of charge carriers across the junction interface region. This study revealed the interfacial upward energy band bending of the stacked Si/GaN heterostructure with surface potentials of 0.99 eV for GaN and 1.14 eV for Si, attributed to the formation of the amorphous interface. These findings challenge the conventional electron affinity model by accounting for interfacial bonding effects. Electrical measurements of the stacked Si/GaN pn heterostructure diode exhibited a rectifying behavior, consistent with the XPS-determined energy band alignment. The diode outperformed early design with a low leakage current density of 5 × 10^−5^ A/cm^2^ and a small ideality factor of 1.22. This work underscores the critical role of the amorphous interface in determining energy band alignment and provides a robust methodology for optimizing the electronic performance of stacked heterostructures. The XPS-based approach can be extended to analyze and develop multi-layered bipolar devices.

## 1. Introduction

Heterogeneous integration using stacking technology offers noteworthy benefits, including overcoming material lattice mismatch issues, achieving multi-layer architectures and inorganic/organic semiconductors, and increasing device power density [1,2,3,4,5,6,7,8,9]. Due to the different physical and chemical properties at the surfaces of individual materials, an interfacial region forms at the heterostructure interface during stacking, which affects the energy band and conduction of carriers across the junction interface.

Transfer-printing crystalline nanomembranes (NMs) on disparate materials facilitates the development of heterogeneously stacked semiconductors, devices, and circuits, enabling the utilization of the advantages of both materials [10,11,12,13,14]. However, the deformation of interface features is inevitably accompanied by material lamination and subsequent junction annealing [15,16]. Despite this, the energy states of stacked semiconductor heterostructures have not been thoroughly explored. The energy band at the stacked interface has typically been assessed using conventional models, without considering the chemical and physical states at the interface [11,17]. The effects of the amorphous interface region formed during the stacking on the energy band alignment of the hybrid heterostructure have not been examined, and the methodology for the assessment has not been established yet.

The band alignment of a core–shell heterostructure nanowire is directly measured using photocurrent spectroscopy [18]. Different from the planar heterostructure interfaces, the local electronic potential is produced along the radial direction of the nanowire. X-ray photoelectron spectroscopy (XPS) can quantify the binding energies (BEs) of atomic core elements at the stacked heterostructure and trace the alterations in BEs along the interface at depths up to few nanometers [19,20]. Since the surface potential of materials can be determined from the changes in BEs, the energy band alignment of a stacked heterostructure can be analyzed using XPS, considering the effects of the amorphous interfacial region.

To date, XPS has been widely employed to determine the band alignments of hybrid heterostructures. However, prior studies have not accounted for the influence of the junction interface on the energy band structure, even though the interface layer is inevitably formed during the fabrication of hybrid heterostructures. This oversight highlights the need for further investigation into the interface layer’s role in shaping the electronic properties of hybrid systems.

In this work, the crystalline Si/GaN heterostructure was formed by NM stacking, and its energy band alignment was analyzed using XPS depth profiling, which considered the effects of the amorphous interfacial layer on the energy band. Si and GaN are representative materials for semiconductor devices and circuits due to the convenience of their growth, doping, and processing and their superior power handling capability [12,21]. Therefore, the formation of a Si/GaN heterostructure enables the integration of the merits of both Si and GaN [11,12,13,22]. Using XPS depth profiling, the energy band alignment of the stacked Si/GaN heterostructure was accurately determined, considering the amorphous interfacial layer. The stacked Si/GaN pn heterostructure diode, whose band alignment was analyzed by XPS depth profiling, exhibited improved electrical performance. Details for the determination of band alignment using XPS are explained in this paper, and the possible application of this methodology to the development of multi-level bipolar devices is suggested.

## 2. Materials and Methods

**Figure 1:**
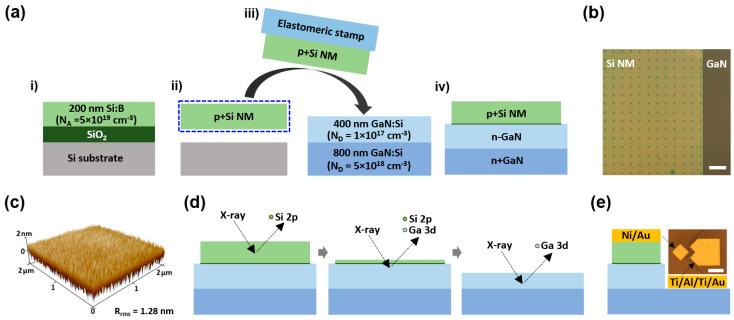
(**a**) Process steps for a stacked Si/GaN heterostructure by nanomembrane (NM) junction bonding: (i) preparation of a highly boron-doped silicon-on-insulator (SOI) wafer, (ii) NM patterning and removal of a sacrificial SiO_2_ layer using HF solution (undercut), (iii) transfer-printing of Si NM onto moderately and highly Si-doped GaN epi-substrate using an elastomeric stamp, and (iv) junction bonding leading to the stacked Si/GaN pn heterostructure. (**b**) Optical top view of the stacked Si/GaN heterostructure. Dark-colored dots on the Si NM surface are etching holes. Scale bar = 100 μm. (**c**) Surface morphology of the GaN epi-substrate, measured by atomic force microscopy. The root mean square surface roughness (R_rms_) is 1.28 nm. (**d**) X-ray photoelectron spectroscopy depth profiling of the stacked Si/GaN heterostructure. Binding energies of core elements are obtained from the top to the bottom through the junction-bonded region. (**e**) Vertical structure of the Si/GaN pn heterostructure diode. The inset image shows the top view of the fabricated device. Scale bar = 50 μm.

**Figure 2:**
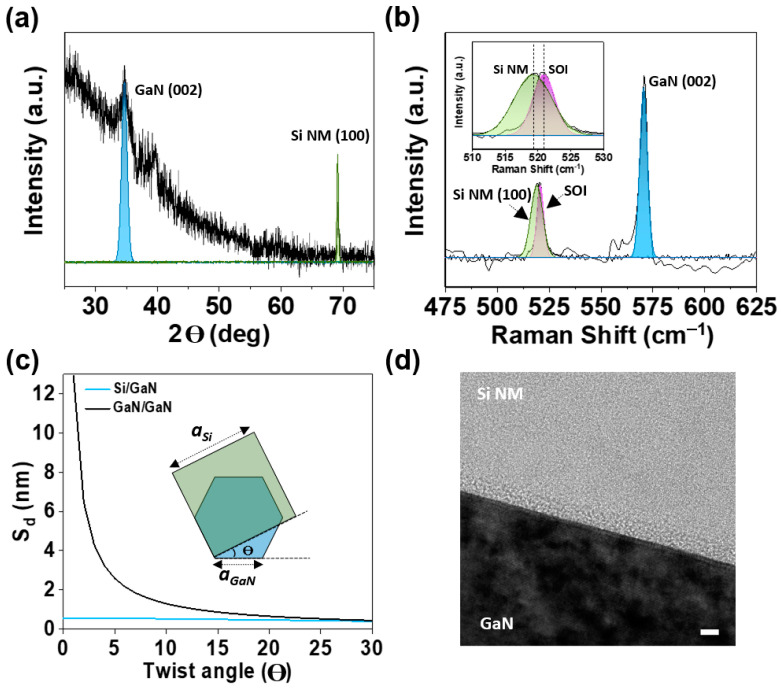
(**a**) Lattice directions of the stacked crystalline Si/GaN heterostructure, analyzed by an X-ray diffractometer. The X-ray was incident to the top surface of Si NM/GaN, resulting in the 2Θ peaks at 69.15° from Si NM (100) and 34.66° from the GaN (002) epi-wafer. (**b**) Raman shifts in the stacked Si/GaN heterostructure with the peaks at 519.5 cm^−1^ from Si NM (100) and 569.4 cm^−1^ from GaN (002). Raman shift in the Si prior to junction bonding (SOI wafer) shows a peak at 521.0 cm^−1^. The inset shows the peak shift in Si NM and SOI. (**c**) Dislocation spacing (S_d_) as a function of twist angle (Θ) between lattice vectors of the stacked heterostructure. *a*_si_ and *a*_Gan_ indicate lattice constants of Si and GaN, respectively. (**d**) High-resolution transmission electron microscopy image of the cross-sectional stacked Si/GaN heterostructure with an amorphous interface region. Scale bar = 2 nm.

The formation of the stacked Si/GaN heterostructure began with the preparation of a highly boron-doped silicon-on-insulator (SOI) wafer (N_A_ = 5 × 10^19^ cm^–3^) (Figure 1a). The thicknesses of the top Si and sacrificial SiO_2_ layers of the SOI were 200 nm and 1 μm, respectively. Etching holes of NM for the undercut were patterned on the surface of SOI with a 5 × 5 μm^2^ size and a 50 μm interval by photolithography and inductively coupled plasma reactive-ion etching (ICP-RIE). The size and interval of the etching holes and the thickness of the SiO_2_ sacrificial layer were determined considering the high undercut rate and high NM formation yield. The undercut was performed by dipping the NM-patterned SOI in 49% HF solution for 10 min to remove the sacrificial layer, resulting in a Si NM/Si substrate. The concentration of the HF solution was chosen to minimize the undercutting time and attack on the Si surface. The Si NM was picked up using an elastomeric polydimethylsiloxane stamp and laminated onto a GaN epi-wafer. The Ga-polar GaN was grown on a sapphire substrate by metal–organic chemical vapor deposition (MOCVD) with doping concentrations of N_D_ = 1 × 10^17^ cm^–3^ for the top 400 nm layer and N_D_ = 5 × 10^18^ cm^–3^ for the bottom 800 nm layer. The relatively low-doped GaN layer formed a heterojunction with the highly doped Si and shaped the depletion region inside GaN. Since the thickness of Si was relatively thin, the low-doped GaN layer was formed to prevent the Si from reaching full depletion (i.e., punch-through). The highly doped GaN layer formed ohmic contact for electrical bias. Junction bonding was conducted at 600 °C for 3 min, resulting in the stacked Si/GaN pn heterostructure. The optical top view of the stacked Si/GaN heterostructure represented arranged etching holes on the Si NM surface, through which the GaN surface was shown (Figure 2b). Surface morphology is an important factor when stacking. The surface roughness of the GaN epi-wafer was measured using atomic force microscopy, leading to 1.28 nm root mean square surface roughness (R_rms_) for a 2 × 2 μm^2^ region (Figure 1c). XPS depth profiling was performed with 1 keV Ar sputtering (Figure 1d). The XPS spectra of core elements (Si 2p, Ga 3d, C 1s, O 2s, and the valence band region) were obtained from the top Si to the bottom GaN through the stacked Si/GaN interface. The Si/GaN pn heterostructure diode was fabricated with the deposition of ohmic contacts of Ni/Au (50/100 nm) for Si and Ti/Al/Ti/Au (5/100/5/100 nm) for GaN, followed by ohmic annealing at 600 °C for 30 s (Figure 1e). The 40 × 40 μm^2^ stacked junction area was defined by ICP-RIE, aligned with the Ni/Au contact (inset).

The GaN epi-wafer was grown by an Aixtron AIX2400G3HT MOCVD reactor. A stacked pn diode was fabricated in a cleanroom facility using a Suss Microtec MA-6 mask aligner, a Sorona SRN-200 e-gun evaporator, and a BMR Hi-Etch ICP etcher. The energy states of atomic core elements were obtained by Thermo Fisher Scientific NEXSA XPS. A cross-sectional image of the stacked heterostructure was captured by JEOL JEM-2100F transmission electron microscopy. Lattice parameters were analyzed by a Bruker D8 DISCOVERY X-ray diffractometer, and surface morphology was measured using PSIA XE150 atomic force microscopy. The Raman shift was obtained using a Horiba LabRam Aramis spectrometer, and electrical characteristics were measured using a Keithley 4200-SCS semiconductor parameter analyzer.

## 3. Results and Discussion

The lattice directions of the stacked crystalline Si/GaN heterostructure were analyzed using an X-ray diffractometer (XRD) (Figure 2a). The X-ray was incident to the top surface of Si NM/GaN, exhibiting 2Θ peaks at 34.66° and 69.15° originated from GaN (002) and Si NM (100). The XRD plot confirmed that the crystalline properties of Si and GaN were maintained after the formation of the stacked Si/GaN heterostructure.

Raman spectroscopy was used to calculate the strain generated during junction bonding (Figure 2b). The Raman shifts in the stacked Si/GaN heterostructure showed peaks at 519.5 cm^−1^ from Si NM (100) and 569.4 cm^−1^ from GaN (002). The Raman shift in the Si prior to the stacking (SOI wafer) showed a peak at 521.0 cm^−1^. The biaxial strains (ε_××_) were estimated as follows:

ε_××_ (%) = −0.136 × Δω (cm^–1^) (1)

where Δω is the difference in Raman shifts. Considering the Δω of Si NM and GaN (with 598 cm^−1^ for unstrained GaN), the biaxial strains were calculated to be 0.20% and 0.19% in Si NM and GaN, respectively [23]. Since the thickness of Si is, relatively, much thinner than that of GaN, the strain generated during junction bonding may exist inside Si. The strain in GaN may originate from the piezoelectric polarization due to the lattice mismatch between GaN and the sapphire substrate during MOCVD growth [10].

Si has a diamond-cubic unit cell with a lattice constant of 5.43 Å, while GaN adopts a Wurtzite structure with a lattice constant of 3.19 Å. The significant differences in their crystal structures and the considerable lattice mismatch resulted in a small dislocation spacing (S_d_) for the Si/GaN heterojunction, defined as

(2) $$S_{d}=\frac{a_{1}\times a_{2}}{\sqrt{2(a_{1}^{2}+a_{2}^{2}\;-2a_{1}\times a_{2}cos\theta )}}$$

where *a*_1_ and *a*_2_ represent the lattice constants of Si and GaN, respectively, and θ is the twist angle between their lattice vectors (Figure 2c) [24]. At a 0° twist angle, the Si/GaN junction exhibited a dislocation spacing of 0.54 nm, which decreased to 0.40 nm at a 30° twist angle. In comparison, the GaN/GaN junction displayed a much larger dislocation spacing of over 13.00 nm at an approximately 0° twist angle, reducing to 0.44 nm at a 30° twist angle. Unlike the GaN/GaN junction, which showed a relatively large dislocation spacing at a low twist angle due to its symmetrical crystal structure, the Si/GaN junction consistently featured small dislocation spacing. This behavior was primarily due to the pronounced lattice mismatch between Si and GaN. Consequently, the Si/GaN heterointerface tended to form an amorphous interlayer which was thermodynamically stable and had a lower energy than that of the crystal structure, aligning the dislocation regions to address the mismatch between the two crystal structures [13].

Atomic-scale properties at the Si/GaN heterostructure interface were examined using high-resolution transmission electron microscopy (HRTEM) (Figure 2d). The HRTEM image shows that Si and GaN are interfaced with a 2 nm amorphous layer region. The amorphous layer may influence the energy band of the stacked Si/GaN heterostructure and affect the charge carrier’s injection at the interface.

**Figure 3:**
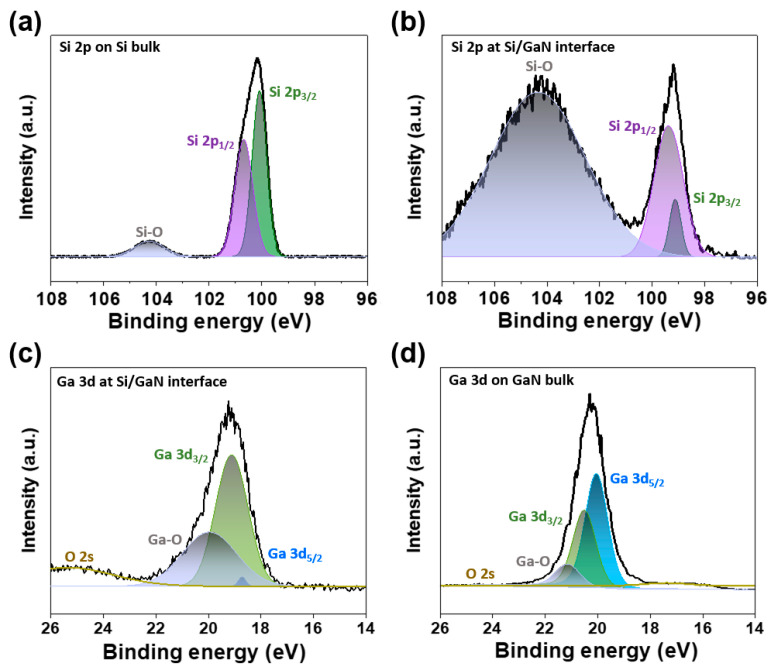
X-ray photoelectron spectroscopy (XPS) spectra of the atomic core elements in the stacked Si/GaN heterostructure, obtained by XPS depth profiling from the top Si to the bottom GaN through the amorphous interface region. Si 2p peaks (**a**) on the Si bulk and (**b**) at the stacked Si/GaN interface. The Si 2p peaks are deconvoluted into Si elements (Si 2p_1/2_ and Si 2p_3/2_) and Si oxide (Si-O) peaks. The asymmetric shapes of the Si 2p peaks are the result of overlapping Si 2p_3/2_ and Si 2p_1/2_ spin–orbit splitting elements. Ga 3d peaks (**c**) at the stacked Si/GaN interface and (**d**) on the GaN bulk. The Ga 3d peaks are deconvoluted into Ga elements (Ga 3d_3/2_ and Ga 3d_5/2_) and Ga oxide (Ga-O) peaks. An O 2s peak is formed in the vicinity of the Ga 3d peak. The asymmetric shapes of the Ga 3d peaks are ascribed to the overlapping of Ga 3d_5/2_ and Ga 3d_3/2_ spin–orbit splitting elements.

XPS depth profiling was used to clarify the energy band alignment of the junction-bonded heterostructure interfaced with the amorphous region. Since XPS can penetrate up to a few nanometers under the surface, it can precisely detect the changes in binding energy and composition of atomic core elements. XPS spectra were obtained every 60 s, etching down the stacked Si/GaN heterostructure from the top Si surface to the bottom GaN substrate through the amorphous interface region. The XPS scan for Si 2p on the top Si surface showed a peak BE of 100.32 eV, while Si 2p at the stacked Si/GaN interface showed a peak BE of 99.18 eV (Figure 3a,b). After deconvolution of the Si 2p peak, using a Gaussian function, into Si oxide (Si-O) and Si element (Si 2p_1/2_ and Si 2p_3/2_) bonds, the area ratios of the Si-O/Si elements were 10.11% on the Si surface and 345.65% at the interface. The asymmetric shape of the Si 2p peaks was the result of overlapping Si 2p_3/2_ and Si 2p_1/2_ elements generated by spin–orbit splitting. The XPS scans for Ga 3d at the interface and on the GaN bulk demonstrated peak BEs of 19.39 eV and 20.28 eV, respectively, with the deconvoluted area ratios of Ga oxide (Ga-O)/Ga elements (Ga 3d_3/2_ and Ga 3d_5/2_) being 67.94% and 16.42% (Figure 3c,d). An XPS O 2s peak was formed in the vicinity of the Ga 3d peak, affecting the asymmetry and peak BEs. The asymmetric shapes of Ga 3d peaks were ascribed to the overlapping of Ga 3d_5/2_ and Ga 3d_3/2_ spin–orbit splitting elements. At the junction interface where the amorphous region was observed, both Si and GaN were oxidized with the oxidation ratio in Si being higher than in GaN. The Gibbs free energy (Δ_f_G°) related to the Si-O and Ga-O bonds was 368 kJ/mol and 285 kJ/mol, respectively, resulting in a higher oxidation ratio in Si at the Si/GaN interface. The experimental parameters of the stacked Si/GaN heterostructure are summarized in Table 1.

**Figure 4:**
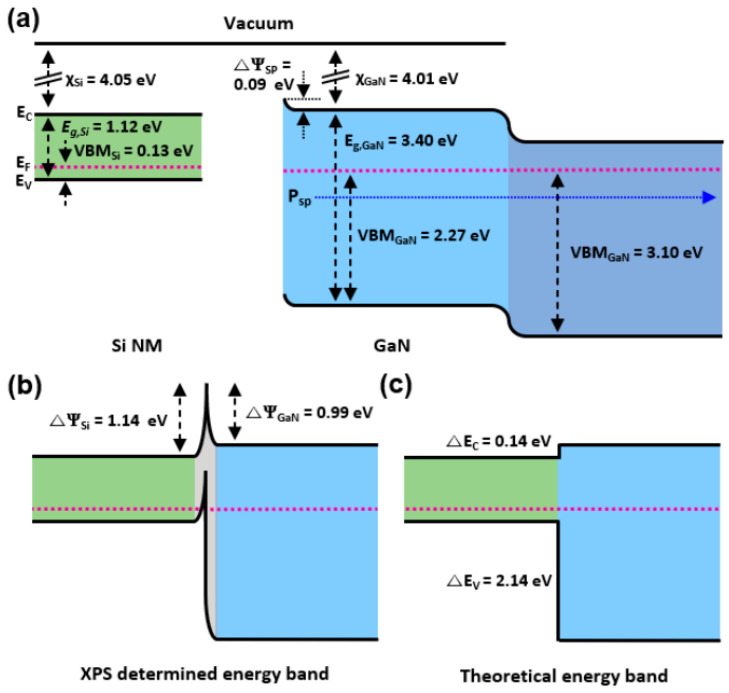
(**a**) Electronic parameters of Si NM and Ga-polar GaN epi-wafer at thermal equilibrium. χ and VMB denote the electron affinity and measured valence band maximum values. P_sp_ and △Ψ indicate spontaneous polarization and surface potential. Upward surface band bending with △Ψ_sp_ took place due to the P_sp_ in the Ga-polar GaN. (**b**) XPS determined the energy band alignment of the stacked Si/GaN heterostructure with an amorphous region, considering the effects of the interface layer formed during junction bonding on the energy. (**c**) Theoretical (electron affinity rule) energy band alignment of Si/GaN with no consideration of the stacked interface region. △E_C_ and △E_V_ represent conduction and valence band offsets, respectively.

The energy band alignment of the stacked Si/GaN heterostructure was depicted using XPS depth profiling, considering the effects of the amorphous interface region on the energy band. The electronic parameters of Si NM and Ga-polar GaN at thermal equilibrium were shown with the band gap (E_g_), electron affinity (χ), and measured valence band maximum (VBM) values (Figure 4a). The VBM values of the Si and GaN surfaces were determined to be 0.13 ± 0.1 eV and 2.27 ± 0.1 eV by the extrapolation of the XPS VBM region, respectively, and the VBM value of the highly doped GaN layer was determined to be 3.10 ± 0.1 eV. The precision error of extrapolation was ±0.1 eV. The x-axis of linear fittings of VBM regions indicated the energy distance to the Fermi level from the VBM. The GaN epi-wafer was etched down to the highly doped region using ICP-RIE for the XPS measurement. The spontaneous polarization (P_sp_ = –0.033 C∙m^−2^) of Ga-polar GaN pointes toward the bulk from the surface, resulting in the negative bound sheet charges on the surface of GaN [21]. The surface potential owing to the spontaneous polarization (△Ψ_sp_) of Ga-polar GaN was 0.09 eV, with upward band bending, determined by the shift in XPS Ga 3d BEs from 20.37 eV (as prepared) to 20.28 eV (as cleaned) [21]. The methodology to determine energy band alignment using XPS was first introduced in previous studies [19,20]. However, the energy band alignment of a stacked heterostructure with an amorphous interface region has not been reported yet. Since the BE indicates the energy difference from the atomic core level to the Fermi level, the surface potential at the interface was calculated by the shift in BEs at the interface and on the bulk. As a result, the band alignment of the stacked Si/GaN heterostructure corresponded to the surface potential changes in the individual Si and GaN at the interface. From XPS depth profiling, the Ga 3d peak shifted from 20.28 eV on the bulk to 19.39 eV at the interface, leading to the upward band bending, with 0.99 eV △Ψ_GaN_ at the stacked interface. The Si 2p peak shifted from 100.32 eV to 99.18 eV, resulting in the upward band bending, with 1.14 eV △Ψ_Si_ at the stacked interface (Figure 4b). The band alignment determined by the XPS depth profiling differed from that obtained via traditional models such as the electron affinity rule which assumes no effect of chemical bonding at the interface (no interface layer) (Figure 4c). Since the interfacial region was unavoidably formed during the stacking of heterogeneous materials, it was necessary to consider the effects of the amorphous interface layer on the entire energy band alignment of the heterostructure, and the method in this work could estimate the effects of the interface region on the energy band alignment of the stacked heterostructure.

**Figure 5:**
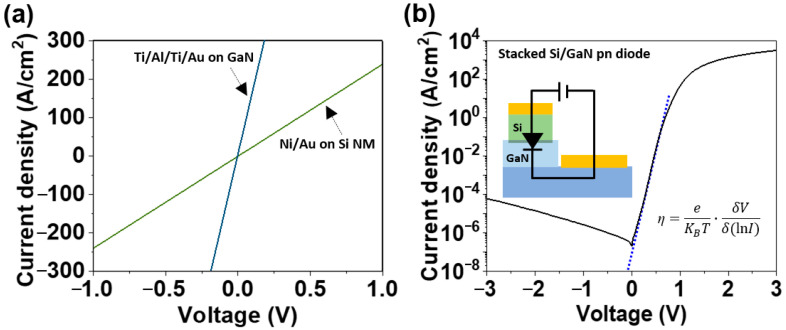
Electrical characteristics of the stacked Si/GaN heterostructure: (**a**) ohmic contact properties of the Ni/Au electrode on the Si NM and Ti/Al/Ti/Au electrode on the GaN and (**b**) rectifying behavior of the stacked pn heterostructure diode, as a basic building block for bipolar electronic devices. *η* is the ideality factor, *e* is the elementary charge, *k* is the Boltzmann constant, and *T* is the temperature.

The electrical characteristics of the stacked Si/GaN heterostructure with an amorphous interface region were investigated to assess the potential application of a stacked pn heterostructure to multi-layered bipolar electronic devices. The Ni/Au electrode on the Si NM and the Ti/Al/Ti/Au electrode on the GaN of the stacked heterostructure exhibited ohmic contact properties, respectively (Figure 5a). The relatively low current density level of Si NM, compared to GaN, was related to the thinness of Si layer. The electrical measurement of the stacked Si/GaN pn heterostructure diode showed a rectifying behavior, with a turn-on voltage around 1 V, corresponding to the surface potential at the stacked Si/GaN interface (Figure 5b). The ideality factor (*η*) of 1.22 was estimated by *η* = *e*/*kT*·δV/δ(ln*I*), where *e* was the elementary charge, *k* was the Boltzmann constant, and *T* was the temperature. The small deviation from the thermionic emission (*η* = 1) was probably due to the interface surface potential. For comparing the electrical properties of a stacked Si/GaN pn heterostructure diode (in this work), previous results of a crystalline Si/GaN photodiode were considered (as a reference) [14]. The device areas for this and the reference work were 1600 μm^2^ and 962.11 μm^2^, respectively. The leakage current density at –3 V was around 5 × 10^−5^ A/cm^2^ and 5 × 10^−4^ A/cm^2^, and the ideality factor was estimated to be 1.22 and 2.60 for this and the reference work, separately. Though the doping concentrations used in this and the reference work were different, junction parameters such as leakage and the ideality factor were improved in the current work. The stacked Si/GaN pn heterostructure diode could be applied to develop bipolar electronic devices such as NPN and PNPN power transistors.

## 4. Conclusions

In conclusion, the energy band alignment of the stacked Si/GaN heterostructure was revealed through XPS depth profiling, emphasizing the impact of the amorphous interface region on the electronic properties. The energy band alignment of the stacked Si/GaN heterostructure exhibited interfacial upward energy band bending with 0.99 eV surface potI ential for GaN and 1.14 eV for Si at the interface, due to the formation of an amorphous interface region during stacking. These findings deviated from the traditional electron affinity model, which neglects interfacial bonding effects. The electrical measurement of the stacked Si/GaN pn heterostructure diode demonstrated a rectifying behavior, corresponding to the energy band alignment determined by XPS. The performance of the stacked Si/GaN pn heterostructure diode surpassed the previously developed one in terms of a low leakage current density (5 × 10^−5^ A/cm^2^) and a small ideality factor (1.22). By addressing the influence of the amorphous interface, this study provides a comprehensive framework for accurately determining the energy band alignment in stacked heterostructures, offering valuable insights for optimizing their electronic performance in advanced applications. The methodology of determining the band alignment of a stacked heterostructure using XPS can be applied to the analysis and development of multi-layered bipolar devices.

## Figures and Tables

**Table 1:** Experimental parameters of the stacked Si/GaN heterostructure determined using XPS.

		Bulk	Interface	VBM at Bulk	Surface Potential at Interface
GaN	Ga 3d BE	20.28 eV	19.39 eV	2.27 ± 0.1 eV (top) 3.10 ± 0.1 eV (bottom)	0.99 eV (△Ga 3d BE)
	Ga-O/Ga element area ratio	10.11%	345.65%		
Si	Si 2p BE	100.32 eV	99.18 eV	0.13 ± 0.1 eV	1.14 eV (△Si 2p BE)
	Si-O/Si element area ratio	16.42%	67.94%		

## Data Availability

The original contributions presented in the study are included in the article, further inquiries can be directed to the corresponding author.
